# Cardiomyocyte Janus kinase 1 (JAK1) signaling is required for cardiac homeostasis and cytokine-dependent activation of STAT3

**DOI:** 10.1016/j.yjmcc.2025.07.017

**Published:** 2025-07-29

**Authors:** Arasakumar Subramani, Kobina Essandoh, Michael Y. Young, Francesca H. Marino, James P. Teuber, Kay-Uwe Wagner, Matthew J. Brody

**Affiliations:** a Department of Pharmacology, University of Michigan, Ann Arbor, MI, USA; b Department of Internal Medicine, University of Michigan, Ann Arbor, MI, USA; c Department of Oncology and Center for Molecular Medicine and Genetics, Wayne State University, Detroit, MI, USA

**Keywords:** Cardiac hypertrophy, Fibrosis, Heart failure, Cardiomyopathy, Cardiomyocyte, Cytokine receptor, JAK, STAT, Intracellular signaling, Oncostatin M

## Abstract

Despite the essential role of inflammation in the pathogenesis of heart failure and other chronic cardiovascular diseases, how cardiomyocytes sense and respond to the inflammatory milieu is not well understood. Cytokine receptors respond to circulating glycoprotein 130 (gp130) family cytokines, such as interleukin-6 (IL-6) and oncostatin M (OSM), by signaling through Janus kinases (JAK) to ultimately elicit phosphorylation-dependent nuclear translocation and transcriptional activity of signal transducer and activator of transcription (STAT) proteins. JAK1 is particularly important for STAT3-dependent cytokine production and macrophage recruitment by cardiomyocytes and STAT3 promotes cardiac hypertrophy and remodeling in response to pressure overload or angiotensin-II but is protective during ischemic injury. However, the roles of JAK1 signaling in cardiac homeostasis and cardiomyocyte cytokine sensing and responsivity remain unclear. To assess the functions of JAK1 in cardiac physiology, we generated mice with cardiomyocyte-specific deletion of JAK1 and evaluated cardiac structure and function, myocardial remodeling, and intracellular signal transduction. Loss of JAK1 in cardiomyocytes results in dilated cardiomyopathy by 6 months of age, indicating cytokine receptor signaling through JAK1 is essential for cardiac physiology. Cardiomyopathy in aged mice lacking cardiomyocyte JAK1 was characterized by substantial myocardial fibrosis. Transcriptomics and gene expression analyses identified JAK1-dependent cytokine-inducible target genes in adult cardiomyocytes as putative effectors of JAK1-STAT3 in the cardiac stress response. JAK1-deficient adult cardiomyocytes were resistant to phosphorylation and nuclear translocation of STAT3 and transcriptional reprogramming in response to OSM. Collectively these data indicate cardiomyocyte JAK1 kinase activity is required for proper cardiac maturation and homeostasis and is indispensable for STAT3 activation and transcriptional responses to OSM.

## 1. Introduction

Cardiomyocytes sense and respond to extracellular hormones, including cytokines, by signaling through transmembrane receptors that activate intracellular signal transduction pathways [1–3]. Type I and type II cytokine receptors activate intracellular signaling through the Janus kinase (JAK)/signal transducer of activator of transcription (STAT) pathway [4,5]. JAK proteins are tyrosine kinases that are at the nexus of transducing signals from extracellular cytokines by phosphorylating the cytokine receptor to elicit binding and recruitment of STAT proteins followed by JAK-mediated phosphorylation of receptor-recruited STAT proteins to induce their dimerization, nuclear translocation, and transcriptional activity [4–7]. JAK-STAT signaling downstream of the class I interleukin-6 (IL-6) family of glycoprotein 130 (gp130)-containing cytokine receptors in response to gp130 family cytokines such as IL-6, leukemia inhibitory factor (LIF), and oncostatin M (OSM), promotes cardiomyocyte hypertrophy and also confers protection of cardiomyocytes from oxidative damage through STAT3 [8–15]. STAT1 and STAT3 are phosphorylated in response to cytokines and angiotensin-II in cardiac myocytes [12,16] although STAT3 signaling is the principal mediator of pathophysiologic adaptation of the heart to circulating cytokines and is required for pathologic cardiac hypertrophy in response to angiotensin-II or pressure overload [13,17–20]. STAT3 signaling in cardiomyocytes plays roles in regulation of the microtubule cytoskeleton during hypertrophic remodeling [21], inflammatory cell recruitment and protection from cell death in response to ischemia [14,15,22–24], transcriptional responses to β-adrenergic stimulation [25], sarcolemmal stability and resistance to viral infection [26], and promotion of angiogenesis and maintenance of myocardial capillary density [9,27,28].

Notably, although JAK1, JAK2, and TYK2 are all activated in cardiomyocytes in response to mechanical stretch and LIF [12,13], gp130 cytokines that induce cardiomyocyte hypertrophy signal primarily through JAK1 and STAT3 [12,29]. Moreover, JAK1-STAT3 signaling specifically in cardiomyocytes is critical for synthesis and secretion of the cytokine Reg3β that recruits macrophages to the mammalian heart in response to myocardial infarction injury [22] and for myocyte proliferation in the regenerating zebrafish heart [30]. Cardiomyocyte-specific deletion of the other major JAK protein in cardiomyocytes, JAK2, results in dilated cardiomyopathy [31] and treatment with a JAK2-specific inhibitor impairs STAT3 phosphorylation and increases apoptosis in rat hearts in response to acute myocardial infarction [8], indicating essential nonredundant functions for JAK2 signaling in cardiac homeostasis and adaptation to ischemic injury. However, the requirement of cardiomyocyte JAK1 signaling in cardiac physiology has not been directly evaluated in vivo.

Here, we tested the role of cardiomyocyte JAK1 signaling in cardiac homeostasis by generating mice with cardiomyocyte-specific deletion of JAK1 and assessing cardiac structure-function, pathophysiology, and cytokine receptor signaling. Loss of cardiomyocyte JAK1 resulted in dilated cardiomyopathy in aged mice characterized by cardiac hypertrophy, impairment of left ventricular systolic function, and myocardial fibrosis. Importantly, adult cardiomyocytes lacking JAK1 were defective in activating STAT3 and transcriptional reprogramming in response to the cytokine OSM, suggesting the oncostatin M receptor (OSMR) couples exclusively to JAK1 in adult cardiomyocytes. These findings demonstrate indispensable functions for JAK1 signaling in postnatal and adult cardiac maturation and/or function but also suggest JAK1-specific inhibitors, which are widely used in adult patients to treat rheumatoid arthritis and myeloproliferative neoplasms and exhibit a relatively safe cardiac toxicity profile [32–34], could potentially be used to attenuate STAT3-dependent transcription in the stressed heart as a means to antagonize adverse cardiac remodeling.

## 2. Materials and methods

### 2.1. Animals

All animal procedures were approved by the University of Michigan Institutional Animal Care and Use Committee (IACUC, Protocol #PRO00010778) and are in compliance with the Guide for the Care and Use of Laboratory Animals (National Institutes of Health). Mice with cardiomyocyte-specific ablation of *Jak1* were generated by crossing conditionally-targeted mice with LoxP sites flanking exon 2 of *Jak1* [35,36] with hemizygous transgenic mice expressing Cre recombinase under control of the α-myosin heavy chain promoter (*Myh6*-Cre, Jackson Strain #011038, B6N.FVB(B6)-Tg(Myh6-cre)2182Mds/J) [37] to elicit recombination of the *Jak1* locus and specific loss of JAK1 in cardiomyocytes. *Jak1* LoxP-targeted mice were backcrossed to C57/Bl6 at least 14 times before crossing to *Myh6*-Cre mice to generate experimental animals on a pure C57/Bl6 genetic background. Genotyping was performed as described previously [35,37] and both sexes of mice were used in all experiments. Echocardiography was performed to evaluate cardiac structure and function in anesthetized mice using a Visualsonics Vevo FT Imaging System (Fujifilm) at the University of Michigan Physiology Phenotyping Core as described previously [38–40].

### 2.2. Adult cardiomyocyte isolation and culture

Cardiomyocytes were isolated from hearts of 8–10 weeks old mice using the Langendorff-free method as described previously [41] and plated for 3–4 h on laminin-coated dishes or 8-well chamber slides (BD Falcon #354108 or Ibidi #80826) in M199 media (Gibco, #11043–023) containing 5 % fetal bovine serum (FBS, Sigma #F4135), 10 mM 2,3-butanedione monoxime (BDM, Sigma, # B0753), and 1 % penicillin-streptomycin (P/S, Hyclone #SV30010) and then put in fresh serum-free M199 with 0.1 % BSA, 10 mM BDM, 1× insulin-transferrin-selenium (ITS, Invitrogen, # 41400045), 1× CD lipid (Gibco, # 11905–031), and 1 % P/S. The next day, cardiomyocytes were washed in PBS and placed in fresh PBS with or without 10 ng/mL oncostatin M (OSM, R&D Systems, #495-MO, diluted from 100 μg/mL stock in PBS with 0.1 % BSA) for 30 min, then washed in PBS and harvested for generation of cell lysates for Western blotting or fixed for immunocytochemistry as described below. Alternatively, for gene expression analyses cardiomyocytes were treated with 10 ng/mL OSM in serum-free M199 media with 0.1 % BSA, 10 mM BDM, 1× ITS, 1× CD lipid for 16 h and then harvested for RNA isolation as described below.

### 2.3. Histology, immunohistochemistry, and immunocytochemistry

Fresh cardiac tissue was fixed in 4 % paraformaldehyde (Electron Microscopy Sciences) diluted in PBS overnight at 4 °C, dehydrated in ethanol, and processed for paraffin embedding, sectioning, and hematoxylin and eosin (H&E) or picrosirius red (PSR) histological staining at the University of Michigan School of Dentistry Core Histology Laboratory to assess gross histopathology and collagen deposition, respectively, as described [42,43]. Paraffin-embedded midventricular transverse cardiac sections were used for histological analyses and immunohistochemistry. Sections were stained with 0.5 μg/mL Alexa-Fluor-conjugated wheat germ agglutinin (Thermo Fisher Scientific, #W11261) to mark the cell membrane and cross-sectional area and minimum Feret’s diameter quantified from 60 cells per animal from images taken of the left ventricular free wall in ImageJ as described previously [43,44]. Interstitial fibrosis was quantified as the mean fraction of PSR-positive area of six 10×-magnification images taken around the left ventricle and perivascular fibrosis quantified as the mean fraction of PSR-positive area in square regions bounding 2 to 3 coronary vessels from images taken of the left ventricular myocardium.

Immunohistochemistry was performed on deparaffinized cardiac sections as described in detail elsewhere [45,46]. Briefly, following deparaffinization and antigen unmasking by boiling in 10 mM sodium citrate pH 6.0 for 5–10 min (Vector Laboratories, # H-3300), sections were blocked and then immunostained with anti-F4/80 (Cell Signaling Technology, #70076S, 1:100) primary antibody in PBS containing 10 % fetal bovine serum (FBS) [45] or blocked and immunostained with anti-PDGFRα (R&D Systems, #AF1062, 1:100) in Pblec buffer (PBS containing 1 % triton X-100, 1 mM MgCl_2_, 1 mM CaCl_2_, and 0.1 mM MnCl_2_) [46]. For F4/80 staining, sections were washed in PBS, incubated in goat anti-rabbit HRP secondary antibody diluted 1:1000 (Thermo Fisher, #31460), washed in PBS, and HRP signal developed for one hour with DAB Substrate kit (Abcam, #ab64238) followed by counterstaining with Harris Hematoxylin (Fisher Scientific, #SH26–500D) and mounting with Permount mounting media (Fisher Scientific, #SP15–100) as described elsewhere [45]. For PDGFRα primary antibody staining, sections were washed and incubated with donkey anti-goat Alexa Fluor 647-conjugated secondary antibody (Thermo Fisher Scientific, #A21447) diluted 1:1000 for 2 h at room temperature, costained with phalloidin conjugated to Alexa Fluor 568 (Thermo Fisher Scientific, #A12380), and nuclei stained and sections mounted with Fluoroshield with DAPI (Sigma, #F6057). Phalloidin-568 was pseudocolored green and anti-PDGFRα staining pseudocolored red for clarity in images in Fig. 5G.

Immunocytochemistry was performed on isolated cardiomyocytes as described [47,48]. Isolated adult cardiomyocytes were fixed in 4 % paraformaldehyde (Electron Microscopy Sciences) diluted in PBS for 15 min at room temperature followed by blocking in immunocytochemistry buffer (PBS, 5 % goat serum, 1 % BSA, 1 % glycine, 0.2 % triton X-100) for one hour at room temperature and incubation with anti-STAT3 (Cell Signaling Technology, #9139, 1:500) primary antibody diluted in immunocytochemistry buffer overnight at 4 °C. After washing in PBS, cells were incubated with goat anti-mouse Alexa Flour 568 secondary antibody (Thero Fisher Scientific, #A11004) diluted 1:1000 in immunocytochemistry buffer for 2 h at room temperature, washed in PBS, and nuclei stained blue with 4′,6-diamidino-2-phenylindole (DAPI, Invitrogen, #D1306). Bright-field imaging of histological staining and F4/80 immunohistochemistry was performed on a Nikon Microphot-SA. Immunofluorescent imaging of cardiac sections and isolated cardiomyocytes was performed on a Zeiss LSM 880 Airyscan confocal microscope. Manders coefficients for colocalization of the anti-STAT3 and DAPI fluorescent signals in isolated cardiomyocytes were quantified using Image J (NIH) as described previously [48] and normalized to 1.0 in the saline-treated control group in Fig. 2F to account for relative staining intensity differences across experiments.

### 2.4. Western blotting

Western blotting was performed as described previously [40,47,49]. Protein lysates were made from cardiac tissue or isolated adult cardiomyocytes in radioimmunoprecipitation assay (RIPA) buffer (50 mM Tris•HCl pH 7.4, 1 % triton X-100, 1 % sodium deoxycholate, 1 mM EDTA, 0.1 % SDS) with protease and phosphatase inhibitor cocktail consisting of 1 mM AEBSF, 10 μg/mL leupeptin, and 10 μg/mL aprotinin and 2 mM Na_3_VO_4_ (Thermo Scientific, #J60191AD). Cardiac tissue was homogenized using the Bullet Blender Tissue Homogenizer (Next Advance) and cell and tissue lysates were centrifuged at 4 °C to pellet insoluble debris and collect the soluble fraction for protein analyses. Immunoblotting was performed by standard SDS-PAGE and transferring of electrophoresed proteins to polyvinylidene difluoride (PVDF) membranes (Millipore Immobilon-FL, #IPVH00010), blocking with 5 % nonfat dry milk diluted in Tris-buffered saline with 0.1 % Tween-20 (TBST) for one hour at room temperature and incubation with primary antibodies diluted in 5 % milk in TBST overnight at 4 °C. Primary antibodies used for immunoblotting were: JAK1 (Sigma, #06–272, 1:500), JAK2 (Cell Signaling Technology, #3230, 1:500), STAT3 (Cell Signaling Technology, #9139, 1:500), p-Y705-STAT3 (Cell Signaling Technology, #9145, 1:500), α-sarcomeric actin (Sigma, #A2172, 1:20,000), PDGFRα (Cell Signaling Technology, #3174, 1:1000) [50], α-tubulin (Santa Cruz, sc-5286, 1:500), and Gapdh (Fitzgerald, #10R-G109A, 1:50,000). PVDF membranes were then washed with TBST, incubated with IRDye-conjugated secondary antibodies (LI-COR Biosciences) diluted 1:10,000 in 5 % milk in TBST with 0.02 % SDS for 1–2 h at room temperature, and then washed again in TBST prior to imaging and quantification of band intensities on an Odyssey CLx scanner (LI-COR Biosciences).

### 2.5. RNA sequencing

RNA was isolated from cultured adult cardiomyocytes with Trizol reagent (Invitrogen), samples pooled and cleaned up with the RNeasy Mini kit (Qiagen, #74104), and RNA sequencing performed on an Illumina NovaSeq PE150 and mapped to the GRCm39/mm39 reference genome (Novogene). FeatureCounts v1.5.0-p3 was used to quantify fragments per kilobase of transcript sequence per million mapped fragments (fpkm) for each gene and identification of differentially expressed genes with a threshold adjusted *P*-value of 0.05 and absolute fold change of 2 performed using the EdgeR R package (3.22.5) [51,52]. KEGG pathway [53] enrichment analysis was performed with Cluster-Profiler R package [54] to identify pathways significantly enriched with differentially upregulated genes in OSM-treated cells (Novogene). The top 10 most-upregulated genes in OSM-treated cells with a minimum expression level of 1.0 fpkm in control vehicle-treated cardiomyocytes are shown in the heat map in Fig. 3C. RNAseq data are available at the NCBI Gene Expression Omnibus (GEO) database with accession number GSE299163.

### 2.6. qPCR

Gene expression was assessed by quantitative real-time polymerase chain reaction (qPCR) as described previously [48]. Total RNA was isolated from ventricular tissue or isolated adult cardiomyocytes using Trizol reagent (Invitrogen) and cDNA synthesis performed with the High-Capacity cDNA Reverse Transcription Kit (Applied Biosystems, 4368814) as described previously [40]. qPCR reactions were performed with synthesized cDNA, specific primers, and PowerUp SYBR Green Master Mix (Applied Biosystems, 4367659) on a QuantStudio 7 Flex qPCR system (Applied Biosystems). Target gene expression was normalized to *Gapdh* expression levels and quantified with the delta-delta Ct method [55]. Sequences of primers used to quantify *Nppa*, *Nppb*, *Myh7*, and *Gapdh* are published elsewhere [38,40] and other primer sequences used are as follows:

*Jak1* For 5′-TGGACAACCGAATAAATGCAGTATC-3′; Rev. 5′-CGGCTGTATACTCTCCGCTG-3′.

*Stat3* For 5′-CTACCCCGACATTCCCAAGG-3′; Rev. 5′-TATTGCTGCAGGTCGTTGGTG-3′.

*Thbd* For 5′- TAGGGCCCTGGATCGGTTTA-3′; Rev. 5′-CTGGTGTGGTTATCGCCAGT-3′.

*Timp1* For 5′- GCAACTCGGACCTGGTCATAA-3′; Rev. 5′-CGCTGGTATAAGGTGGTCTCG-3′.

*Socs3* For 5′-GGAGATTTCGCTTCGGGACT-3′; Rev. 5′-TCGCTTTTGGAGCTGAAGGT-3′.

*Sprr1a* For 5′-GCTCTTCTCTGAGTATTAGGACCA-3′; Rev. 5′-GTTTTGGGGGCACAAGGTTC-3′.

*Serpinb1a* For 5′-ACCTGCTAAGCAAGAGACTTCA-3′; Rev. 5′-AGATGTTTCCTGTGGGGCTG-3′.

*Vegfa* For 5′-GGCCTCCGAAACCATGAAC-3′; Rev. 5′-GCCTGGGACCACTTGGC-3′.

*Postn* For 5′-CTGCTTCAGGGAGACACACC-3′; Rev. 5′-TCTGGCCTCTGGGTTTTCAC-3′

*Nox4* For 5′-CCAAATGTTGGGCGATTGTGT-3′; Rev. 5′-CAGGACTGTCCGGCACATAG-3′.

*Ccl2* For 5′- AGCCAACTCTCACTGAAGCC-3′; Rev. 5′-GCGTTAACTGCATCTGGCTG-3′.

### 2.7. Statistical analyses

Data in histograms are presented as the mean value ± the standard error of the mean. Statistical analyses were conducted using GraphPad Prism 10 and significance testing was performed as described in the figure legends.

## 3. Results

### 3.1. Generation of Jak1 conditional deletion mice

**Fig. 1.**
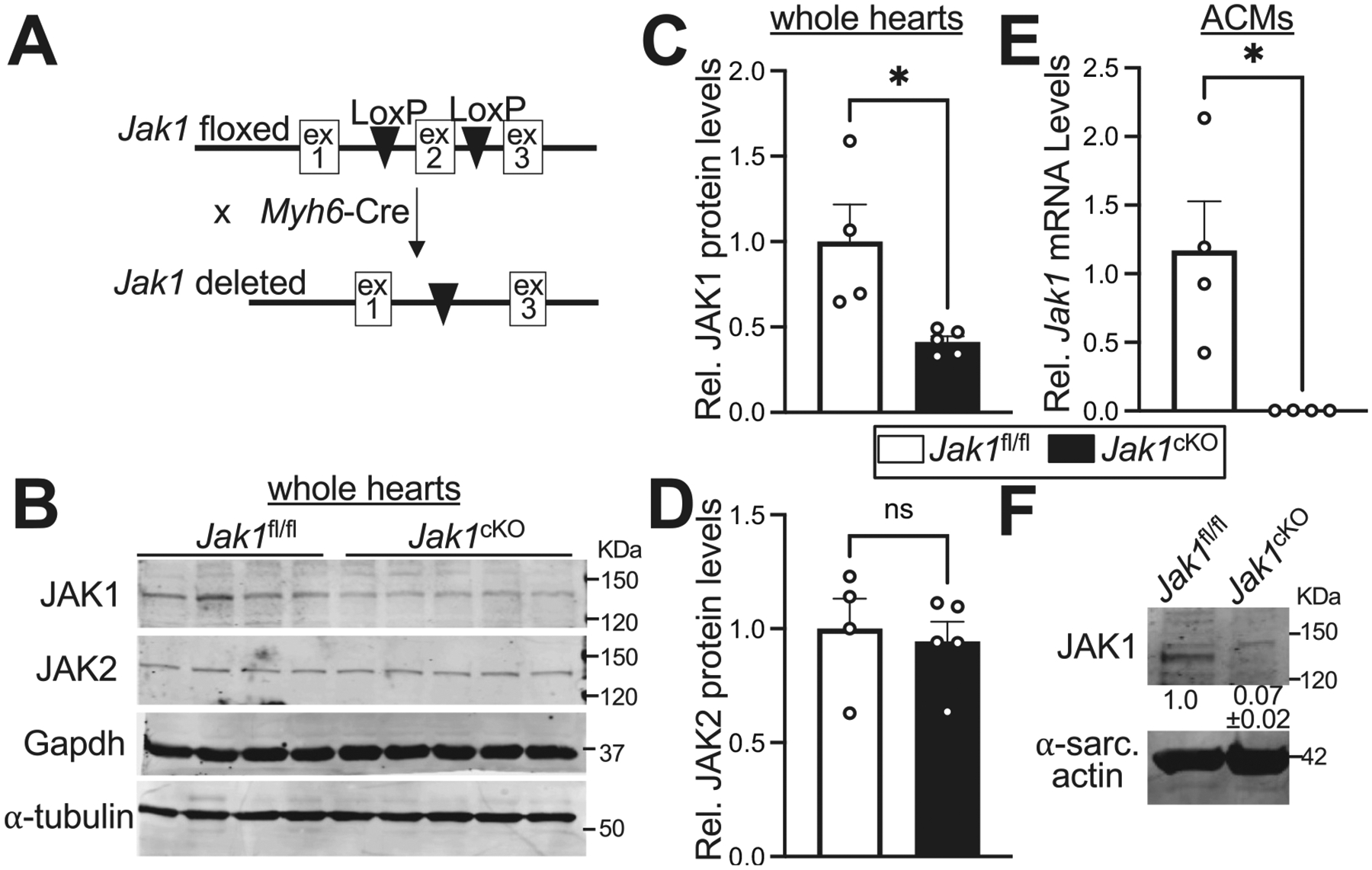
Cardiomyocyte-specific deletion of *Jak1*. (A) Schematic of breeding scheme to generate mice lacking JAK1 in cardiomyocytes by crossing mice harboring a conditional null allele for *Jak1* to α-MHC-Cre (*Myh6*-Cre) hemizygous transgenic mice to produce *Jak1*^fl/fl, *Myh6*-Cre^ (*Jak1*^cKO^) mice with *Jak1* deleted in cardiomyocytes. (B) Immunoblotting and quantification of relative (C) JAK1 and (D) JAK2 protein levels in whole cardiac lysates generated from the indicated genotypes of male and female mice at two months of age. *n* = 4 *Jak1*^fl/fl^ and 5 *Jak1*^cKO^. JAK protein levels were normalized to Gapdh as a loading control. Assessment of (E) *Jak1* mRNA levels by qPCR and (F) JAK1 protein levels by immunoblotting in adult cardiomyocytes (ACMs) isolated from hearts of 2 months old male and female mice. n = 4 in E and *n* = 3 in F. α-sarcomeric (sarc.) actin was used as a loading control for normalization of relative JAK1 protein levels shown on the immunoblotting panel in F. Data are presented as the mean value ± the standard error of the mean. *P < 0.05, *ns*, not significant, unpaired *t*-test.

To interrogate the functions of JAK1 signaling in adult cardiomyocytes, we generated mice with cardiomyocyte-specific loss of JAK1 by crossing mice containing a conditional null *Jak1* allele with LoxP sites flanking exon 2 [35,36] to transgenic mice expressing Cre recombinase driven by the α-myosin heavy chain (α-MHC) promoter (*Myh6*-Cre) [37] (Fig. 1A). Immunoblotting total cardiac lysates revealed a significant reduction in JAK1 protein levels (Fig. 1B, C) but no alteration in JAK2 protein levels (Fig. 1B, D) in hearts of *Jak1*^fl/fl; *Myh6*-Cre^ (*Jak1*^cKO^) mice compared to *Jak1*^fl/fl^ controls. Isolation of adult cardiomyocytes followed by qPCR and Western blotting revealed a complete loss of *Jak1* mRNA (Fig. 1E) and JAK1 protein (Fig. 1F) expression, respectively, in *Jak1*^cKO^ cardiomyocytes, confirming ablation of JAK1.

### 3.2. Defective STAT3 signaling in JAK1-deficient cardiomyocytes

**Fig. 2.**
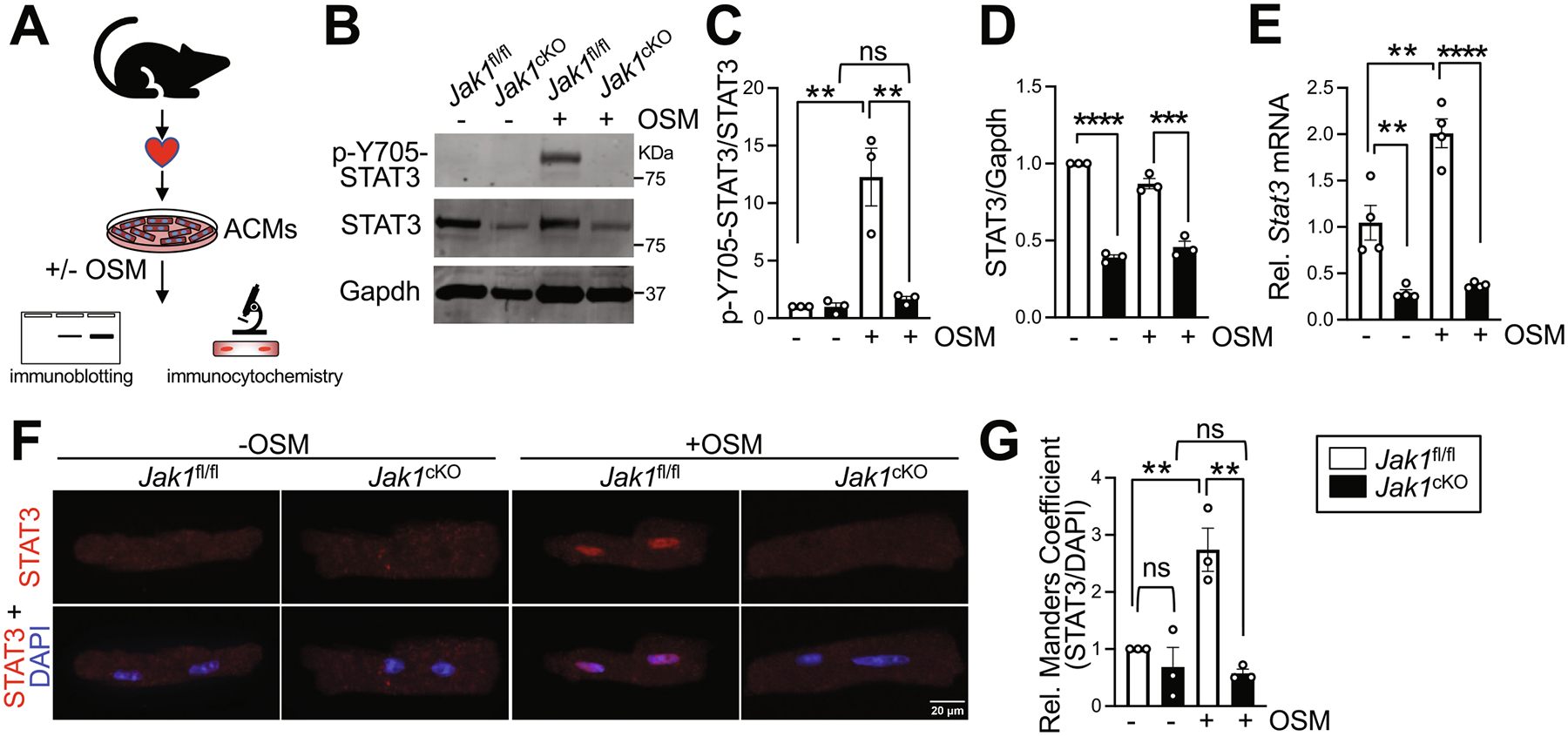
Defective STAT3 signaling in *Jak1*-deleted cardiomyocytes. (A) Experimental assessment of JAK-STAT signaling in *Jak1*-deleted cardiomyocytes. Adult ventricular cardiomyocytes (ACMs) were isolated from male and female control (*Jak1*^fl/fl^) or *Jak1*-deleted (*Jak1*^cKO^) hearts at 2 months of age and treated with vehicle or oncostatin-M (OSM, 10 ng/mL) for 30 min before harvesting for Western blotting or fixing for immunocytochemistry to assess STAT3 phosphorylation and nuclear translocation, respectively, or for 16 h to assess gene expression. (B) Western blotting and quantification of (C) STAT3 phosphorylation at Tyr-705 normalized to total STAT3 and (D) total STAT3 protein normalized to Gapdh in control *Jak1*^fl/fl^ or *Jak1*^cKO^ ACMs with or without OSM treatment. *n* = 3. (E) *Stat3* mRNA levels were quantified by qPCR in ACMs isolated from *Jak1*^fl/fl^ or *Jak1*^cKO^ hearts. *n* = 4. (F) Representative images of immunostaining for endogenous STAT3 (red) in control (*Jak1*^fl/fl^) or *Jak1*-deleted (*Jak1*^cKO^) ACMs with or without OSM treatment. Nuclei were stained blue with DAPI. Scale bar = 20 μm. (G) Relative Manders coefficients for colocalization of STAT3 with nuclear DAPI signal in ACMs of the indicated genotype and treatment. n = 3 independent experiments with 35–60 ACMs analyzed per biological replicate. Data are presented as the mean value ± the standard error of the mean. **P < 0.01, ***P < 0.001, ****P < 0.0001, *ns*, not significant, two-way ANOVA with post-hoc Tukey’s multiple comparisons test. Also see Supplemental Fig. S1.

To evaluate the necessity for JAK1 for STAT3 activation and transcriptional activity, we isolated and cultured adult ventricular cardiomyocytes (ACMs) from *Jak1*^fl/fl^ control or *Jak1*^cKO^ mice for assessment of STAT3 phosphorylation and nuclear translocation in response to stimulation with the cytokine OSM (Fig. 2A). Immunoblotting revealed that *Jak1*-deleted ACMs were completely resistant to phosphorylation of STAT3 in response to OSM (Fig. 2B, C), indicating JAK1 is required for activation of STAT3 in response to OSMR stimulation. Total STAT3 protein levels were also substantially reduced in *Jak1*-deleted ACMs regardless of stimulation (Fig. 2B, D), concomitant with downregulation of *Stat3* transcript levels (Fig. 2E), suggesting some potential feedback mechanism to downregulate STAT3 in the absence of JAK1. Moreover, *Stat3* mRNA levels were significantly upregulated in control ACMs in response to OSM (Fig. 2E). Immunocytochemistry revealed robust nuclear translocation of STAT3 in response to OSM in *Jak1*^fl/fl^ control ACMs whereas *Jak1*-deleted ACMs were completely resistant to OSM-induced STAT3 nuclear translocation (Fig. 2F, G). Importantly, ACMs isolated from *Myh6*-Cre transgenic control mice exhibited robust phosphorylation and nuclear translocation of STAT3 in response to OSM stimulation (Supplemental Fig. S1A–D), indicating loss of JAK1 underlies defective STAT3 signaling and that JAK1 has an indispensable role in cytokine-stimulated activation of STAT3 in adult cardiomyocytes.

### 3.3. JAK1 is required for cytokine-induced transcriptional reprogramming in cardiomyocytes

**Fig. 3.**
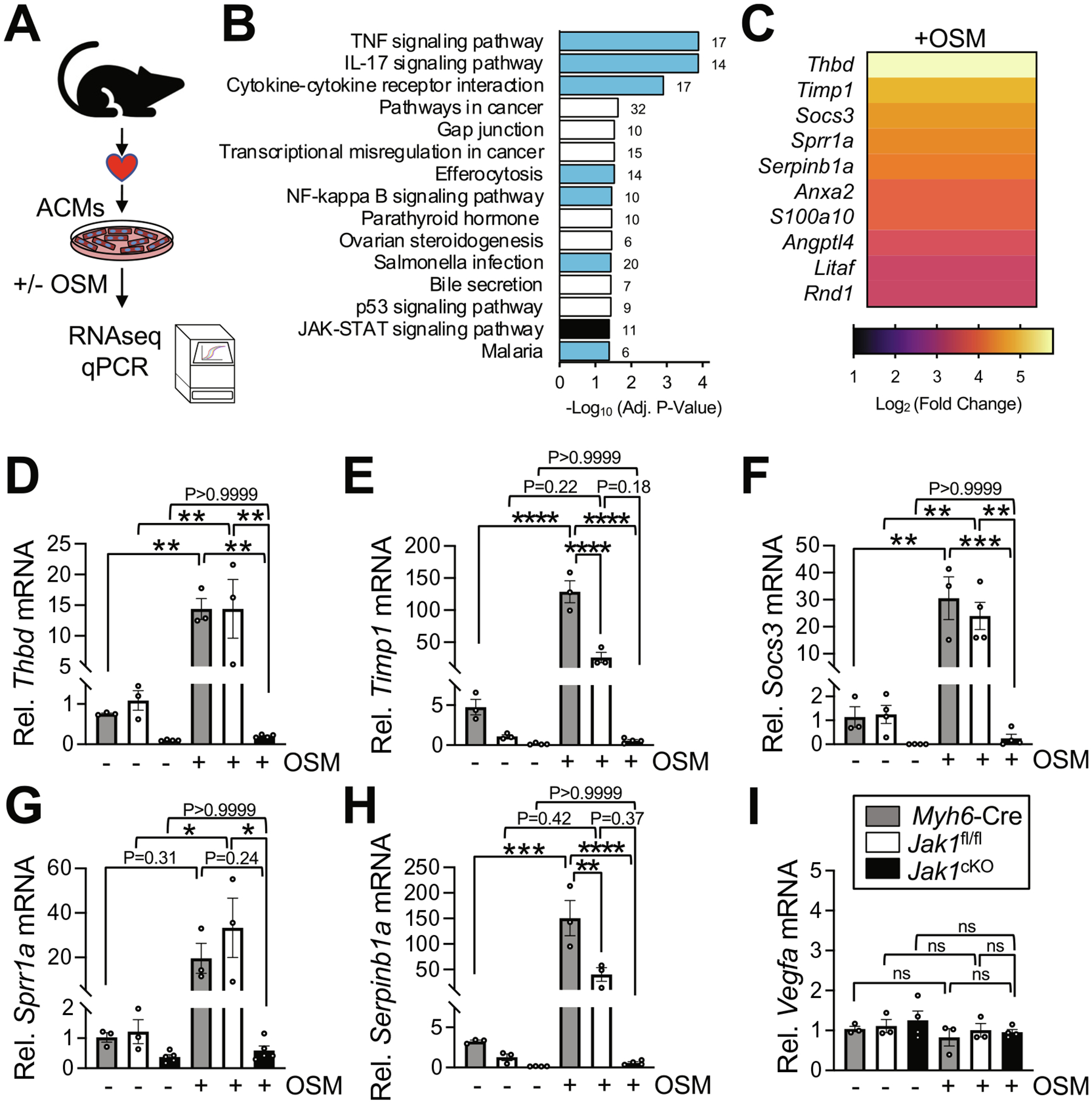
JAK1 is required for cytokine-stimulated transcriptional reprogramming in cardiac myocytes. (A) Experimental assessment of oncostatin-M (OSM)-inducible target genes. Adult ventricular cardiomyocytes (ACMs) isolated from male and female mice were treated with vehicle or OSM (10 ng/mL) for 16 h in culture, and RNA isolated for (B—C) RNA sequencing (RNAseq) or (D—H) qPCR. (B) Histogram of the top 10 KEGG biological processes most significantly enriched with upregulated genes in OSM-treated ACMs compared to vehicle-treated ACMs. Values at the end of histograms indicate the number of upregulated genes with OSM treatment in the pathway. Histograms for biological processes related to inflammation are colored light blue and the JAK-STAT pathway itself colored black. (C) Heat map depicting the 10 most-upregulated transcripts in OSM-treated ACMs relative to vehicle-treated ACMs by RNAseq. Relative mRNA levels of (D) *Thbd*, (E) *Timp1* (F) *Socs3*, (G) *Sprr1a*, (H) *Serpinb1a*, and (I) *Vegfa* were assessed by qPCR in ACMs isolated from male and female mice of the indicated genotypes and treated with vehicle or OSM. n = 3 *Myh6*-Cre, 3 *Jak1*^fl/fl^, and 4 *Jak1*^cKO^ in D, E, H, and I; n = 3 *Myh6*-Cre, 4 *Jak1*^fl/fl^, and 4 *Jak1*^cKO^ in F; n = 3 *Myh6*-Cre, 3 *Jak1*^fl/fl^, and 5 *Jak1*^cKO^ in G. Data are presented as the mean value ± the standard error of the mean. *P < 0.05, **P < 0.01, ***P < 0.001, ****P < 0.0001, *ns*, not significant, two-way ANOVA with post-hoc Tukey’s multiple comparisons test.

Given the remarkable necessity of JAK1 for phosphorylation and nuclear translocation of STAT3 in ACMs in response to OSM stimulation (Fig. 2B, C, F, G), we set out to determine the role of JAK1 in STAT3-dependent transcription and cytokine-induced transcriptional reprograming. To identify physiologic transcriptional targets activated downstream of OSM and putative effectors of JAK1-STAT3 signaling in myocytes, we treated ACMs in culture with OSM and performed RNA sequencing (RNAseq) (Fig. 3A). Most notably, unbiased KEGG pathway analysis revealed a significant enrichment of upregulated genes in OSM-treated ACMs in biological processes related to inflammation and immune response (Fig. 3B). Amongst the most upregulated genes in ACMs in response to OSM treatment was *Socs3* (Fig. 3C), a negative feedback regulator of JAK-STAT signaling and prototypical hallmark cytokine-inducible STAT3 target gene [56,57]. There were relatively few downregulated genes in OSM-stimulated ACMs with most downregulated transcripts predicted to be noncoding (Supplemental Data File 1). Many of the genes most substantially upregulated by OSM stimulation, including *Socs3*, are involved in inflammation, fibrosis, or tissue injury responses (Fig. 3C, Supplemental Data File 1). Most robustly induced by OSM treatment in ACMs were transcripts encoding thrombomodulin, a vascular endothelial cell surface glycoprotein involved in coagulation whose mRNA levels have been reported to be induced in cardiomyocytes by mechanical stretch [58], and TIMP-1, a matrix metalloproteinase inhibitor associated with fibrotic remodeling in the heart [59,60]. OSM stimulation of ACMs also potently induced transcripts encoding small proline-rich protein 1A (SPRR1A), which is upregulated in cardiomyocytes by the gp130 family cytokine LIF and promotes cardiomyocyte apoptosis and maladaptive remodeling in response to myocardial infarction [61,62], and SerpinB1, a serine protease inhibitor involved in immune responses [63].

To determine if JAK1 is required for transcriptional reprogramming of these target genes in cardiomyocytes in response to OSM stimulation, we evaluated mRNA levels of these top 5 OSM-induced genes by qPCR in ACMs isolated from hearts of *Jak1*^cKO^ mice and *Myh6*-Cre or *Jak1*^fl/fl^ controls with or without OSM stimulation. Remarkably, while transcript levels of *Thbd* (Fig. 3D), *Timp1* (Fig. 3E), *Socs3* (Fig. 3F), *Sprr1a* (Fig. 3G), and *Serpinb1a* (Fig. 3H) were all increased orders of magnitude by OSM treatment in ACMs isolated from *Myh6*-Cre and *Jak1*^fl/fl^ control hearts, *Jak1*^cKO^ ACMs were refractory to upregulation of these target genes, indicating JAK1 is required for OSM-stimulated transcriptional responses. Importantly, transcript levels for vascular endothelial growth factor (*Vegfa*), a STAT3 target gene in cardiomyocytes [9,27] that was not induced by OSM treatment in ACMs (Supplemental File 1), were not altered in ACMs by OSM treatment or loss of JAK1 as assessed by qPCR (Fig. 3I), revealing specificity for JAK1 kinase activity in facilitating STAT3-dependent transcriptional reprogramming of cardiomyocytes in response to OSM. Moreover, although studies herein were not sufficiently powered to detect a significant difference, all of the OSM-inducible target genes tested had lower expression levels in unstimulated *Jak1*^cKO^ ACMs compared to *Myh6*-Cre and *Jak1*^fl/fl^ control ACMs in all biological replicates assayed (Fig. 3D–H), suggesting potential JAK1-dependent regulation of these transcriptional targets in cardiomyocytes in the absence of cytokine stimulation as well.

### 3.4. Cardiomyocyte-specific loss of JAK1 results in dilated cardiomyopathy

**Fig. 4.**
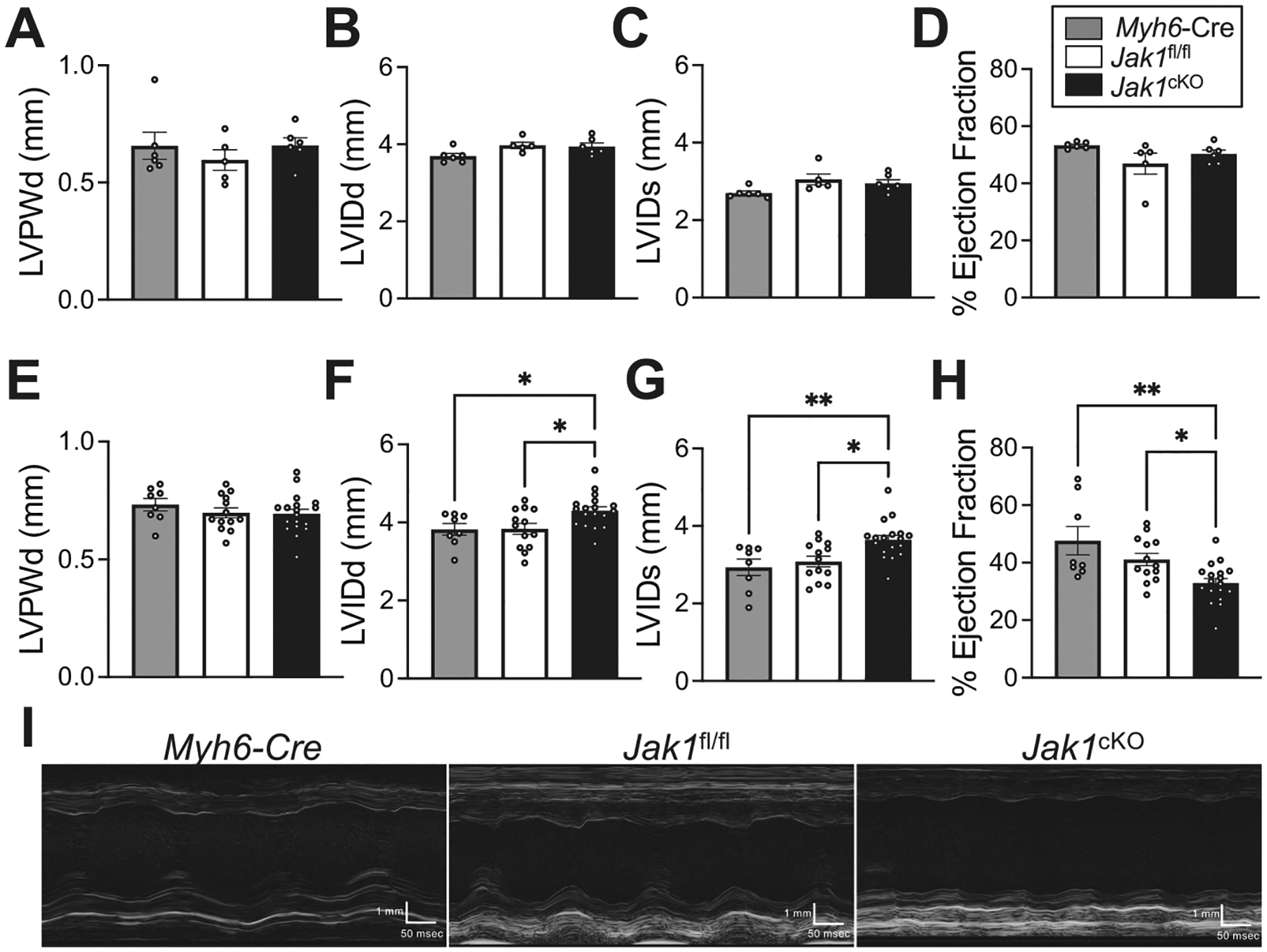
Cardiomyocyte-specific loss of JAK1 results in dilated cardiomyopathy. (A-K) Assessment of cardiac structure and function in conditional-null *Jak1* mice with cardiomyocyte-specific loss of JAK1 (*Jak1*^fl/fl, *Myh6*-Cre^, *Jak1*^cKO^) compared to control genotypes expressing the conditional-null allele (*Jak1*^fl/fl^) or *Jak1*^+/+, *Myh6*-Cre^ mice expressing the Cre transgene alone (*Myh6*-Cre) at (A-D) 2 months and (*E*-H) 6 months of age. Quantification of (A, E) diastolic left ventricular (LV) posterior wall thickness (LVPWd), (B, F) LV internal diameter (LVID) during diastole (LVIDd), (C, G) systolic LVID (LVIDs), and percent (D, H) ejection fraction (E.F.) in the indicated genotypes of mice of both male and female mice. (I) Representative M-mode images of the indicated genotypes of male mice at 6 months of age. *n* = 6 *Myh6*-Cre, 5 *Jak1*^fl/fl^, and 6 *Jak1*^cKO^ in A-D and *n* = 8 *Myh6*-Cre, 13 *Jak1*^fl/fl^, and 18 *Jak1*^cKO^ in E-H. Data are presented as the mean value ± the standard error of the mean. *P < 0.05, **P < 0.01, one-way ANOVA with post-hoc Tukey’s multiple comparisons test. Also see Supplemental Table S1 and Table S2.

**Fig. 5.**
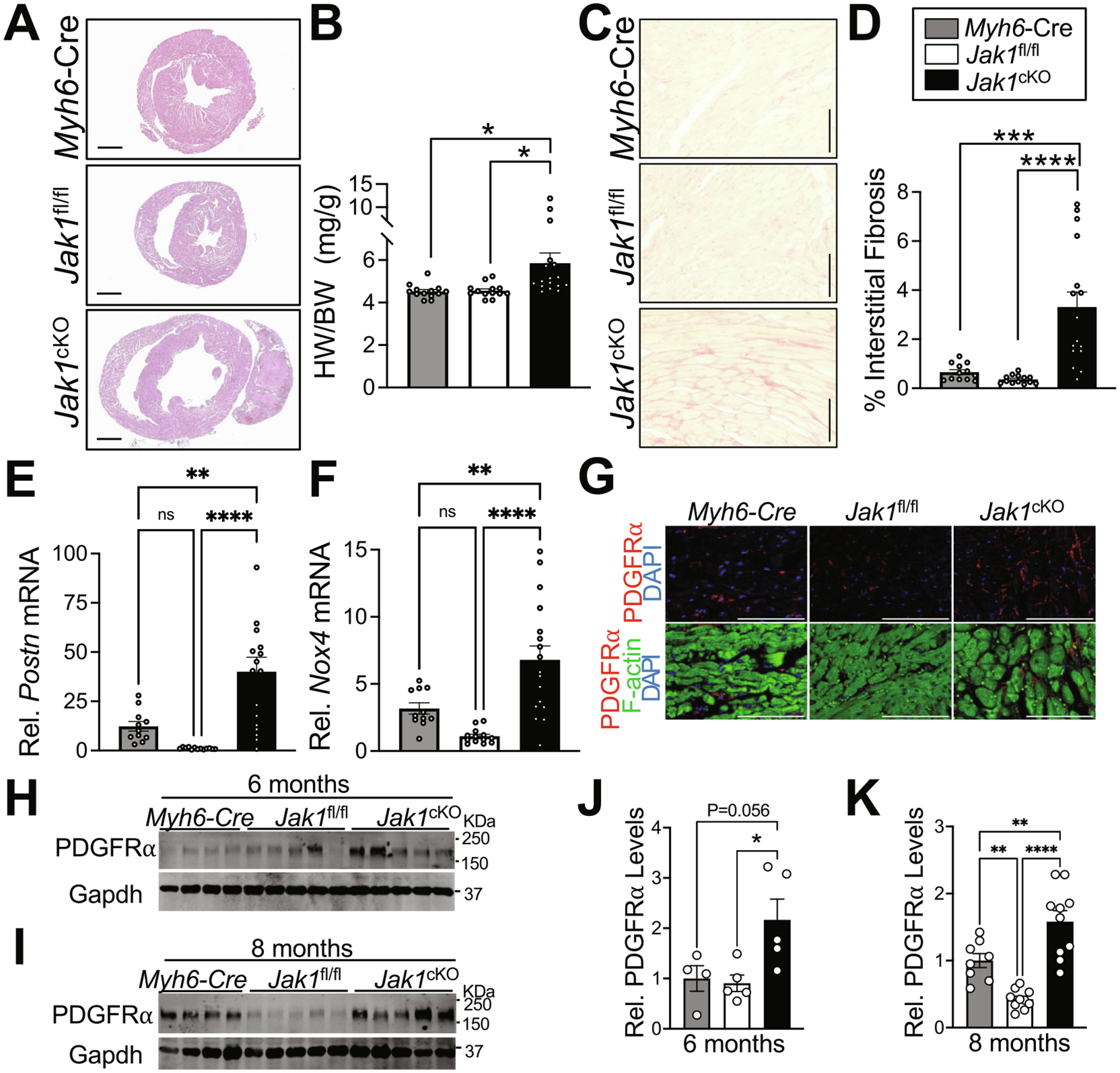
Pathological cardiac hypertrophy and myocardial fibrosis in aged mice with cardiomyocyte-specific loss of JAK1. (A) H&E histological staining of cardiac sections (scale bar = 1 mm) of male mice and (B) heart weight-to-body weight ratios (HW/BW) of the indicated genotypes of male and female mice at 8 months of age. *n* = 13 *Myh6*-Cre, 13 *Jak1*^fl/fl^, and 17 *Jak1*^cKO^. (C) Representative images of Picro Sirius Red-stained cardiac sections (scale bar = 100 μm) and (D) quantification of interstitial myocardial fibrosis in male and female mice of the indicated genotypes at 8 months of age. *n* = 12 *Myh6*-Cre, 13 *Jak1*^fl/fl^, and 16 *Jak1*^cKO^. Representative images in C are mixed sexes (*Myh6*-Cre male, *Jak1*^fl/fl^ and *Jak1*^cKO^ female). Cardiac transcript levels of (E) *Postn* and (F) *Nox4* quantified by qPCR in male and female mice. *n* = 11 *Myh6*-Cre, 13 *Jak1*^fl/fl^, and 17 *Jak1*^cKO^. (G) Representative images of immunohistochemistry for the fibroblast marker platelet-derived growth factor receptor-α (PDGFRα) (red) in cardiac sections of 8 months old male mice. Cardiomyocytes were stained with phalloidin (F-actin, green) and nuclei stained with DAPI (blue). Scale bar = 100 μm. (H, I) Western blotting and (J, K) quantification of PDGFRα protein levels in hearts of the indicated genotypes of male and female mice at (H, J) 6 months and (I, K) 8 months of age. Gapdh was used as a loading control. n = 4 *Myh6*-Cre, 5 *Jak1*^fl/fl^, and 5 *Jak1*^cKO^ in J and 8 *Myh6*-Cre, 10 *Jak1*^fl/fl^, and 10 *Jak1*^cKO^ in K. Data are presented as the mean value ± the standard error of the mean. *P < 0.05, **P < 0.01, ***P < 0.001, ****P < 0.0001, *ns*, not significant, one-way ANOVA with post-hoc Tukey’s multiple comparisons test. Also see Supplemental Figs. S3 and S4.

To determine the necessity of JAK1 for cardiac homeostasis, we evaluated cardiac-structure function by serial echocardiography in *Jak1*^cKO^ mice with cardiomyocyte-specific loss of JAK1 compared to *Jak1*^fl/fl^ controls and *Jak1*^+/+; *Myh6*-Cre^ (*Myh6*-Cre) control mice containing the Cre transgene but not the LoxP-targeted *Jak1* allele. *Jak1*^cKO^ mice are phenotypically normal at 2 months of age (Fig. 4A–D, Supplemental Table S1) but develop dilated cardiomyopathy by 6 months of age (Fig. 4E–I, Supplemental Table S2). No changes in left ventricular (LV) wall thickness were observed in *Jak1*^cKO^ mice compared to control genotypes (Fig. 4A, E) but LV dilation occurred by 6 months of age (Fig. 4F) and was accompanied by significant impairment of systolic function (Fig. 4G–I, Supplemental Table S2), revealing dilated cardiomyopathy in aged mice with cardiomyocyte-specific loss of JAK1. JAK2 protein levels were not altered in hearts of *Jak1*^cKO^ mice at 6 months of age (Supplemental Fig. S2) when functional decompensation is observed due to *Jak1* deletion (Fig. 4). H&E-staining of cardiac sections revealed substantial cardiac enlargement and LV dilation in *Jak1*^cKO^ mice at 8 months of age (Fig. 5A). Cardiac hypertrophy was confirmed in *Jak1* cardiomyocyte-deleted mice at 8 months of age (Fig. 5B, Supplemental Fig. S3A–D).

*Myh6*-Cre transgenic control mice used here are known to develop a cardiac phenotype with age [64]. Although the dilated cardiomyopathy phenotype observed with cardiomyocyte loss of JAK1 was not present in *Myh6*-Cre controls at 6 months of age (Fig. 4, Supplemental Table S2) nor was cardiac hypertrophy observed at the whole organ level at 8 months of age (Fig. 5B, Supplemental Fig. S3B) compared to *Jak1*^fl/fl^ controls, we did observe an increase in cardiomyocyte size in *Myh6*-Cre hearts at 8 months of age, albeit not to the same extent as cardiomyocytes of *Jak1*-deleted hearts (Supplemental Fig. S3A, C–D). Molecular markers of pathologic cardiac growth, such as expression of the hypertrophic marker genes *Myh7*, *Nppa*, and *Nppb* (Supplemental Fig. S3E–G) were similarly upregulated in *Myh6*-Cre transgenic and *Jak1*^cKO^ hearts compared to *Jak1*^fl/fl^ controls, indicating Cre toxicity drives part of the maladaptive cardiac phenotype observed in *Jak1*^cKO^ mice.

### 3.5. Interstitial fibrosis in hearts of aged mice lacking cardiomyocyte JAK1

To further probe cardiac pathology and adverse cardiac remodeling due to cardiomyocyte-specific loss of JAK1 we examined cardiac fibrosis in aged *Jak1* conditionally-deleted mice. Picrosirius red (PSR) staining of cardiac sections revealed substantial interstitial cardiac fibrosis in *Jak1*^cKO^ mice at 8 months of age not observed in control genotypes (Fig. 5C, D). Perivascular fibrosis, however, was not altered in the heart by cardiomyocyte deletion of *Jak1* at 8 months of age (Supplemental Fig. S4A, B). Upregulation of the myofibroblast marker *Postn* [65] (Fig. 5E), and the cardiac fibroblast-enriched gene *Nox4* [66] (Fig. 5F) was also observed in aged *Jak1*-deleted hearts. Immunohistochemistry for the cardiac fibroblast marker platelet-derived growth factor (PDGFRα) [50] in 8 months old mice revealed fibroblast expansion in *Jak1*^cKO^ hearts relative to controls (Fig. 5G). Cardiac fibroblast content was more carefully probed in *Jak1*-conditionally deleted hearts by immunoblotting for PDGFRα, revealing elevated PDGFR*α* protein levels in aged hearts lacking cardiomyocyte JAK1 (Fig. 5H–K). The *Myh6*-Cre transgene has been reported to induce cardiac fibrosis with aging [64] and although here we did not observe significant alterations in cardiac fibrosis in *Myh6*-Cre mice by histochemical PSR staining compared to *Jak1*^fl/fl^ controls at 8 months of age (Fig. 5C, D, Supplemental Fig. S4A, B), we did observe increased PDGFRα protein levels in *Myh6*-Cre transgenic hearts at 8 months of age compared to *Jak1*^fl/fl^ controls, albeit to a significantly lesser extent than *Jak1*^cKO^ hearts (Fig. 5I, K). Importantly, PDGFRα protein levels were significantly induced in *Jak1*^cKO^ hearts at 6 months of age prior to any observed increase of PDGFRα levels in *Myh6*-Cre transgenic hearts (Fig. 5H, J), indicating loss of JAK1 accelerates cardiac fibroblast expansion and fibrotic remodeling in comparison to expression of the *Myh6*-Cre transgene alone. By 8 months of age, *Myh6*-Cre transgenic hearts did exhibit increased PDGFRα protein levels compared to *Jak1*^fl/fl^ controls, however induction of PDGFRα levels was significantly greater yet in *Jak1*^cKO^ hearts (Fig. 5G, I, K). Markers of inflammation, such as F4/80^+^ macrophages as detected by immunohistochemistry (Supplemental Fig. S5A, B) and transcript levels of the chemokine *Ccl2* (Supplemental Fig. S5C) were similarly elevated in *Myh6*-Cre and *Jak1*^cKO^ hearts compared to *Jak1*^fl/fl^ controls at 8 months of age, suggesting inflammation in aged *Jak1*^cKO^ hearts is largely due to cardiotoxicity from chronic cardiomyocyte expression of Cre recombinase [64]. Taken together, these data indicate chronic loss of JAK1 in cardiac myocytes results in cardiac maladaptation characterized by systolic dysfunction, dilative remodeling, and interstitial fibrosis.

## 4. Discussion

We report that JAK1 signaling downstream of sarcolemmal cytokine receptors is required for proper physiology and function of the adult heart. Germline deletion of *Jak1* in mice is perinatally lethal [67] precluding analysis of the role of JAK1 in the adult heart. Here we found that perinatal loss of JAK1 in cardiomyocytes is detrimental, with dilated cardiomyopathy developing in cardiomyocyte *Jak1*-deleted mice by 6 months of age. Cardiomyopathy in *Jak1*-deficient hearts is characterized by left ventricular dilation, systolic dysfunction, and substantial cardiac fibrosis. Indispensable roles for JAK1 in cardiomyocyte signaling are not unexpected given the preponderance of evidence supporting roles for type I and type II cytokine receptors and signaling through STAT3 in cardiac physiology and adaptation to stress. Mice with whole-body deletion of OSMR or cardiomyocyte-specific loss of the gp130 cytokine receptor in cardiomyocytes develop severe dilated cardiomyopathy in response to pressure overload [68,69]. Transgenic overexpression of STAT3 in cardiomyocytes results in cardiac hypertrophy but confers protection from cardiomyopathy in response to doxorubicin [10] whereas loss of STAT3 causes dilated cardiomyopathy in aged mice and exacerbates doxorubicin-induced cardiotoxicity [70]. Thus, cytokine receptor signaling through JAK1 in the unstressed heart and downstream STAT3-dependent transcriptional programs in cardiomyocytes, even in the absence of overt stress or myocardial injury, are essential for proper cardiac homeostasis.

Conditional deletion of *Jak2* in cardiomyocytes results in dilated cardiomyopathy [31], similar to the phenotype reported here for conditional loss of *Jak1*, indicating indispensable and nonredundant functions for both JAK1 and JAK2 in cardiac myocytes. Indeed, JAK1 and JAK2 could have nonoverlapping functions in transducing signals from distinct cytokine receptors in cardiomyocytes to encode specificity for activation of STAT3-dependent transcription in response to unique cytokines in the circulation. Notably, cardiomyocyte deletion of *Jak2* resulted in a reduction of STAT3 phosphorylation as detected in total cardiac lysates of unstressed adult mice [31] whereas with deletion of *Jak1* we observed a reduction in total STAT3 levels in cardiomyocytes at baseline and complete abrogation of STAT3 phosphorylation, nuclear translocation, and induction of target genes in response to OSM. Thus, JAK2 may be responsible for more tonic cytokine receptor signaling and basal activation of STAT3 in cardiomyocytes while JAK1 may mediate more adaptive STAT3 signaling in response to increasing circulating levels of stress-inducible cytokines such as OSM or IL-6.

Consistent with a stress-responsive role for JAK1 kinase activity in cardiomyocytes, we identified novel OSM-inducible genes in adult cardiac myocytes as potential effectors of JAK1-STAT3 in cardiac stress adaptation. Moreover, JAK1 but not JAK2 is essential for STAT3-dependent transcription, synthesis, and secretion of the cytokine Reg3β by cardiomyocytes in response to OSM that aids in the recruitment of immune cells to the infarcted heart [22]. These data collectively suggest JAK1 is necessary for the activation of STAT3 signaling and transcriptional reprogramming in adult cardiomyocytes in response to OSM, which is elevated in the circulation of heart failure patients [71,72] and is critical for adaptation of the heart to acute injury and chronic stress [69,73]. Here, we demonstrate a requisite role for JAK1 activity in cardiomyocyte sensing and responding to OSM that is predominantly released into the circulation by immune cells [74], which has implications for intercellular communication and cardiac adaptation not only to myocardial infarction and heart failure but also the cardiac response to a myriad of chronic diseases and other conditions associated with inflammation.

OSMR and JAK1 signaling play critical roles in the developing heart and in cardiomyocyte dedifferentiation and proliferative capacity [30,73,75] as well that could contribute to the DCM phenotype we observe in adult mice with perinatal loss of cardiomyocyte JAK1. Indeed, OSMR signaling maintains cardiomyocytes in a dedifferentiated state that renders them more plastic and amenable to cell cycle reentry in the developing and injured heart [30,73,75] and aberrant cardiomyocyte maturation due a deficit in JAK1 signaling could impact postnatal cardiac development and maturation that could be cardiomyopathic in adulthood. In the context of cardiac hypertrophy, inhibition of STAT3 signaling is generally protective [13,17–20], suggesting antagonizing JAK1 signaling in adulthood may even be antihypertrophic and cardioprotective. Significantly, OSMR deletion protects from dilated cardiomyopathy in muscle LIM protein (MLP) knockout mice [73] which, coupled with data herein demonstrating an explicit requirement for JAK1 in cytokine-induced STAT3 activation, nuclear translocation, and transcriptional reprogramming, suggests JAK1 signaling contributes to maladaptation in certain forms of heart disease. Notably, although JAK1 is indispensable for induction of OSM-inducible target genes in ACMs such as *Socs3*, *Timp1*, and *Sprr1a*, JAK1 is not required to maintain expression of *Vegfa*, a STAT3 target gene induced by the gp130 cytokine LIF in cardiomyocytes [9,27], nor is *Vegfa* expression increased by OSM treatment, suggesting specificity of gp130 family cytokine receptor signaling in coupling to specific JAK proteins and downstream STAT3-dependent gene programs. All together, these data uncover JAK1 activity as a nexus in cardiomyocyte sensing OSM and transducing intracellular signaling to elicit transcription of target genes that likely contribute to cardiac stress adaptation and remodeling of the heart in the presence of circulating cytokines. JAK inhibitors are widely used to treat autoimmune and inflammatory conditions, most notably rheumatoid arthritis [6], as well as to alleviate cytokine storm in critically-ill COVID19 patients [6,76–78], suggesting treatment with JAK1 specific inhibitors may even attenuate STAT3-dependent hypertrophy, cytokine production, and immune cell recruitment that could alleviate excessive inflammation and reduce adverse cardiac remodeling in the stressed adult heart. Future studies with inducible cell-type specific deletion of *Jak1* in adult mice will help uncover the translational potential of targeting JAK1 in cardiac hypertrophy and failure.

## Supplementary Material

Supplemental Material S1 Uncropped Blots

Supplemental Table S1

Supplemental Table S2

Supplemental Material S2 Figs S1-S5

Supplemental Material S3

Supplementary data to this article can be found online at https://doi.org/10.1016/j.yjmcc.2025.07.017.

## Data Availability

The data that support the findings, including statistical analyses, and reagents used are available from the corresponding author upon request.
