# Supplementary figures and images for: Cardiomyocyte Janus kinase 1 (JAK1) signaling is required for cardiac homeostasis and cytokine-dependent activation of STAT3
Source: J Mol Cell Cardiol. Author manuscript; Available in PMC 2025 Aug 22. (PMC12370008; doi:10.1016/j.yjmcc.2025.07.017)

Fig 1B

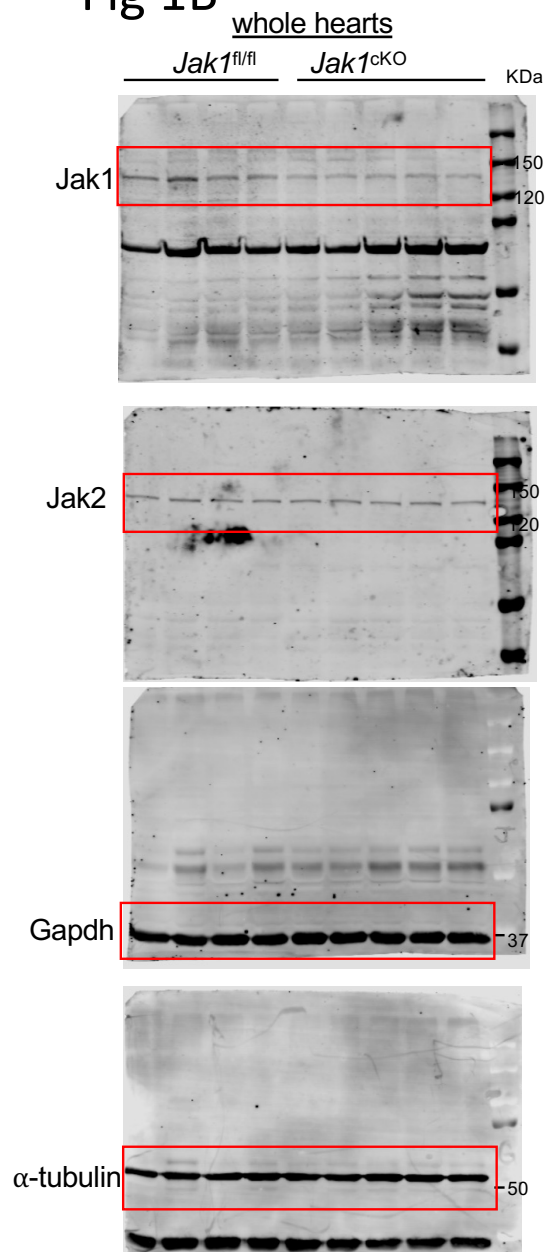

Fig 1F

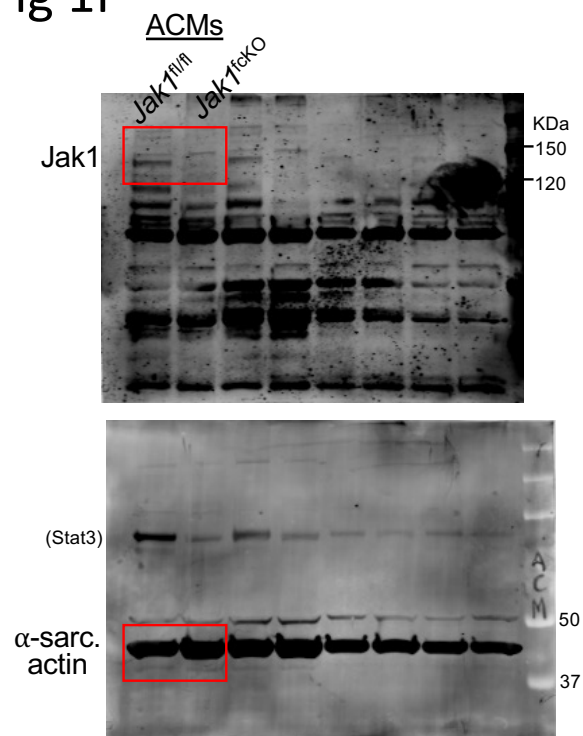

Fig 2B

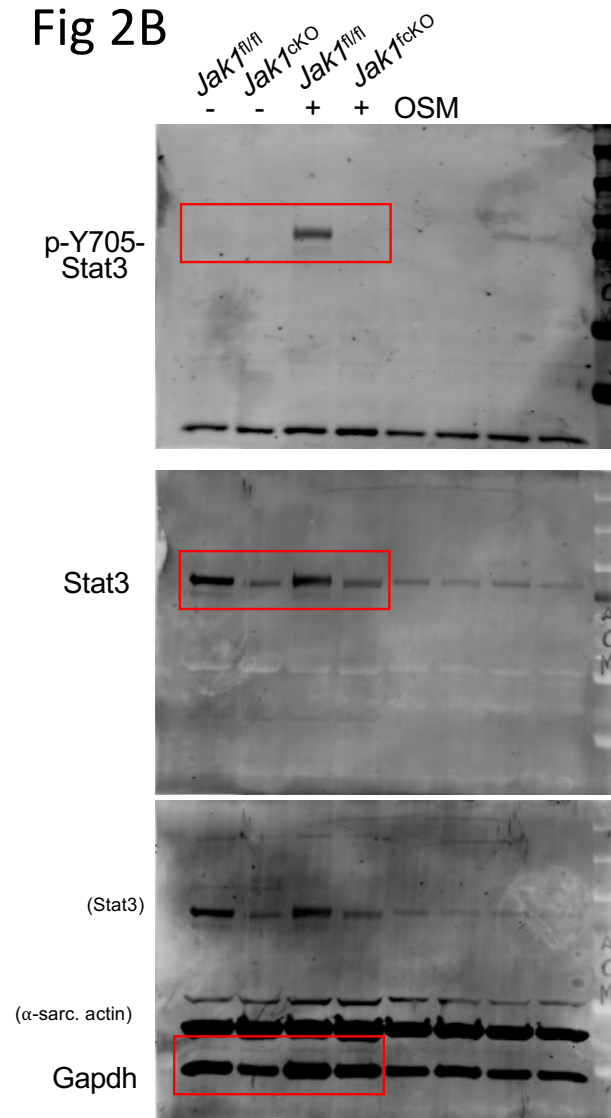

Fig 5H

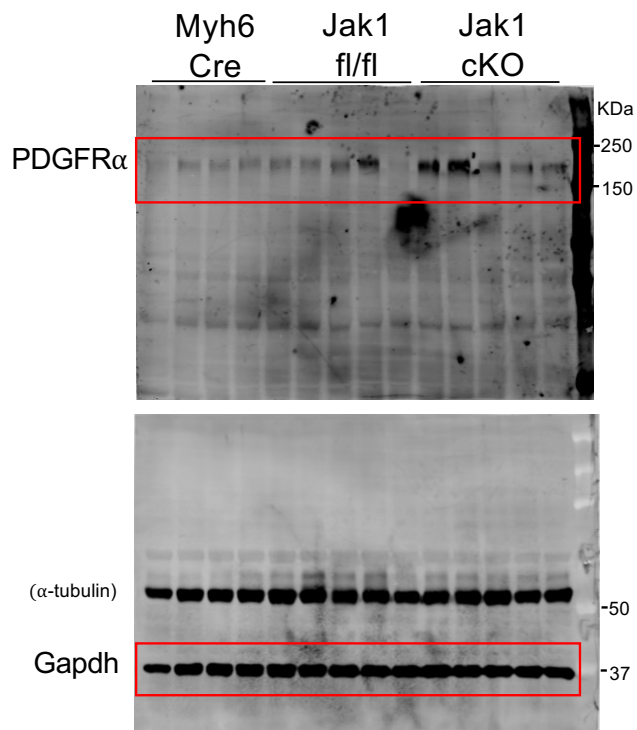

Fig S1A

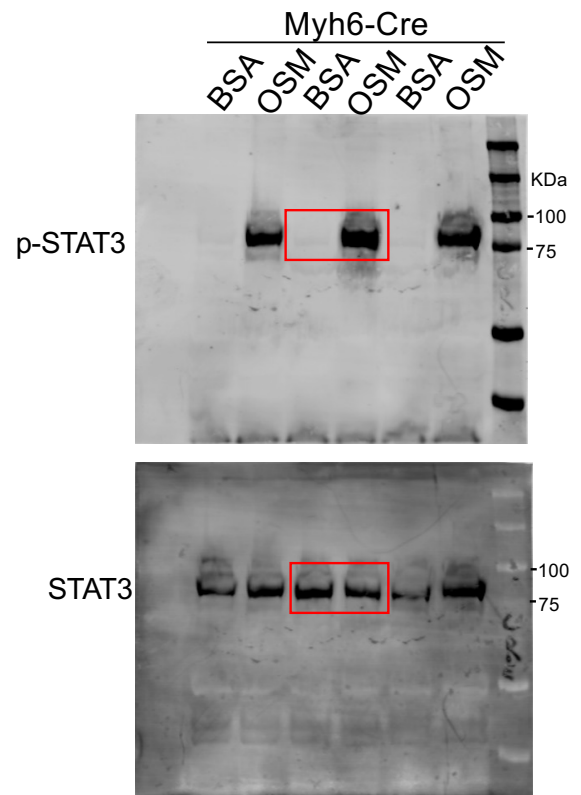

Fig 5I

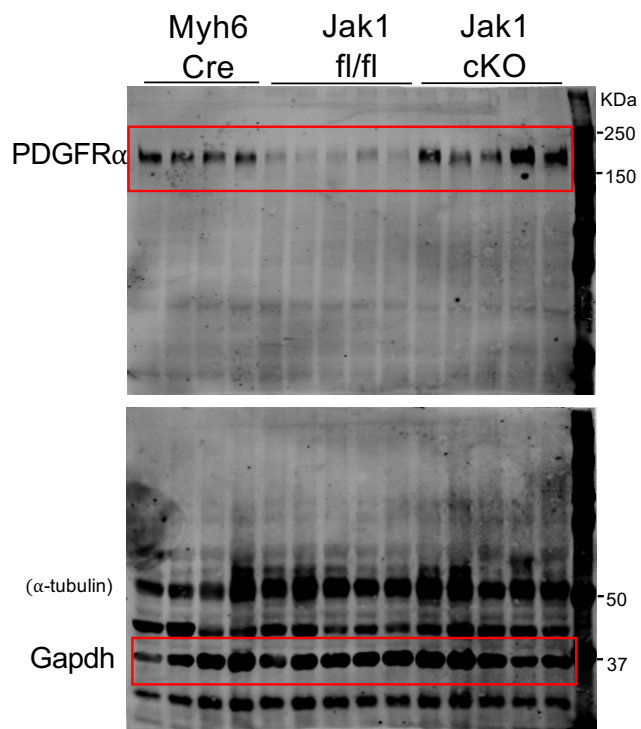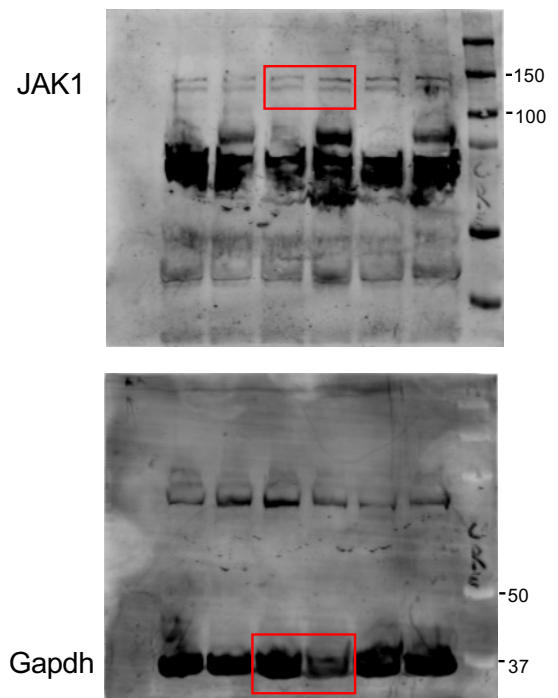

Fig S2A

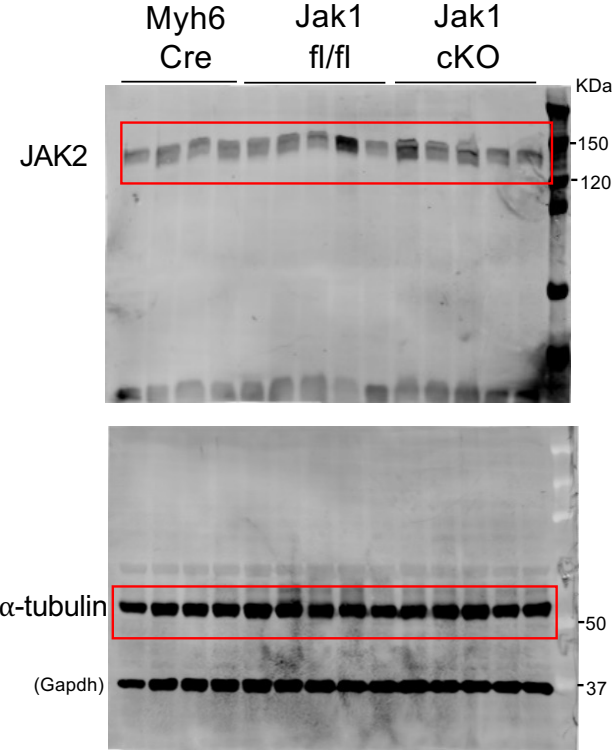

Supplement: Supplemental Material S1 Uncropped Blots [file NIHMS2104056-supplement-Supplemental_Material_S1_Uncropped_Blots.pdf]
