# Supplementary material for: Cardiomyocyte Janus kinase 1 (JAK1) signaling is required for cardiac homeostasis and cytokine-dependent activation of STAT3
Source: J Mol Cell Cardiol. Author manuscript; Available in PMC 2025 Aug 22. (PMC12370008; doi:10.1016/j.yjmcc.2025.07.017)
Supplement: Supplemental Table S1 [file NIHMS2104056-supplement-Supplemental_Table_S1.docx]

|  | *Myh6*-Cre | *Jak1* ^fl/fl^ | *Jak1*^cKO^ |
| --- | --- | --- | --- |
| Number of mice per group | 6 | 5 | 6 |
| LVIDd, mm | 3.69±0.17 | 3.97±0.18 | 3.94±0.22 |
| LVPWd, mm | 0.66±0.14 | 0.60±0.10 | 0.66±0.08 |
| LVAWd, mm | 0.76±0.08 | 0.69±0.04 | 0.66±0.04^#^ |
| LVIDs, mm | 2.70±0.13 | 3.05±0.32 | 2.95±0.23 |
| LVPWs, mm | 0.87±0.16 | 0.88±0.02 | 0.87±0.10 |
| LVAWs, mm | 1.10±0.11 | 0.99±0.08 | 0.99±0.07 |
| LV volume at diastole, μL | 57.98±6.68 | 69.05±7.51 | 67.95±9.13 |
| LV volume at systole, μL | 27.09±3.25 | 37.12±10.07 | 34.01±6.65 |
| Ejection fraction, % | 53.29±1.09 | 46.90±8.20 | 50.28±3.26 |
| Fractional shortening, % | 26.89±0.68 | 23.26±4.61 | 25.15±1.91 |
| LV mass, mg | 87.53±14.05 | 87.88±16.13 | 89.49±12.97 |
| LV mass/body weight, mg/g | 4.26±0.38 | 3.94±0.99 | 4.20±0.69 |
| Heart rate, beats/min | 314.2±28.6^^^ | 449.6±54.3 | 417.0±37.9^##^ |
| BW, g | 20.50±2.45 | 22.58±2.42 | 21.55±3.25 |

**Table S1.** Echocardiographic assessment of cardiac structure-function in mice with cardiomyocyte-specific ablation of *Jak1* at 2 months of age. LVID, left ventricular (LV) inner diameter; LVPW, LV posterior wall; LVAW, LV anterior wall; d, diastolic; s, systolic; BW, body weight. Data are presented as mean ± standard deviation. *P<0.05, **P<0.01 *Jak1*^fl/fl^ compared to *Jak1*^fl/fl;^ *^Myh6^*^-Cre^ (*Jak1*^cKO^) and ^#^P< 0.05, ^##^P< 0.01 *Myh6*-Cre compared to *Jak1*^cKO^, ^^^P<0.001 *Myh6*-Cre compared to *Jak1*^fl/fl^*,* one-way ANOVA with Tukey’s multiple comparisons test.
