# Supplementary material for: Cardiomyocyte Janus kinase 1 (JAK1) signaling is required for cardiac homeostasis and cytokine-dependent activation of STAT3
Source: J Mol Cell Cardiol. Author manuscript; Available in PMC 2025 Aug 22. (PMC12370008; doi:10.1016/j.yjmcc.2025.07.017)
Supplement: Supplemental Table S2 [file NIHMS2104056-supplement-Supplemental_Table_S2.docx]

|  | *Myh6*-Cre | *Jak1* ^fl/fl^ | *Jak1*^cKO^ |
| --- | --- | --- | --- |
| Number of mice per group | 8 | 13 | 18 |
| LVIDd, mm | 3.82±0.42 | 3.84±0.51 | 4.30±0.43*^#^ |
| LVPWd, mm | 0.73±0.07 | 0.70±0.08 | 0.69±0.08 |
| LVAWd, mm | 0.80±0.08 | 0.83±0.07 | 0.78±0.10 |
| LVIDs, mm | 2.93±0.60 | 3.08±0.49 | 3.64±0.49*^##^ |
| LVPWs, mm | 0.98±0.14 | 0.90±0.10 | 0.83±0.13^#^ |
| LVAWs, mm | 1.08±0.13 | 1.10±0.11 | 1.00±0.11 |
| LV volume at diastole, μL | 64.10±15.92 | 65.17±19.83 | 84.42±20.05*^#^ |
| LV volume at systole, μL | 35.17±15.14 | 38.96±14.43 | 57.66±19.31*^#^ |
| Ejection fraction, % | 47.63±13.95 | 41.09±7.66 | 32.91±6.84*^##^ |
| Fractional shortening, % | 24.01±8.63 | 19.82±4.25 | 15.52±3.49^##^ |
| LV mass, mg | 103.45±16.49 | 105.10±24.40 | 120.07±22.21 |
| LV mass/body weight, mg/g | 4.14±0.53 | 3.78±0.59 | 4.37±0.42** |
| Heart rate, beats/min | 463.4±63.5 | 455.4±33.2 | 482.5±58.4 |
| BW, g | 25.14±3.84 | 27.58±3.99 | 27.49±4.57 |

**Table S2.** Echocardiographic assessment of cardiac structure-function in mice with cardiomyocyte-specific ablation of *Jak1* at 6 months of age. LVID, left ventricular (LV) inner diameter; LVPW, LV posterior wall; LVAW, LV anterior wall; d, diastolic; s, systolic; BW, body weight. Data are presented as mean ± standard deviation. *P<0.05, **P<0.01 *Jak1*^fl/fl^ compared to *Jak1*^fl/fl;^ *^Myh6^*^-Cre^ (*Jak1*^cKO^) and ^#^P< 0.05, ^##^P< 0.01 *Myh6*-Cre compared to *Jak1*^cKO^*,* one-way ANOVA with Tukey’s multiple comparisons test.
