# Supplementary material for: Cardiomyocyte Janus kinase 1 (JAK1) signaling is required for cardiac homeostasis and cytokine-dependent activation of STAT3
Source: J Mol Cell Cardiol. Author manuscript; Available in PMC 2025 Aug 22. (PMC12370008; doi:10.1016/j.yjmcc.2025.07.017)
Supplement: Supplemental Material S2 Figs S1-S5 [file NIHMS2104056-supplement-Supplemental_Material_S2_Figs_S1-S5.pdf]

Fig S1

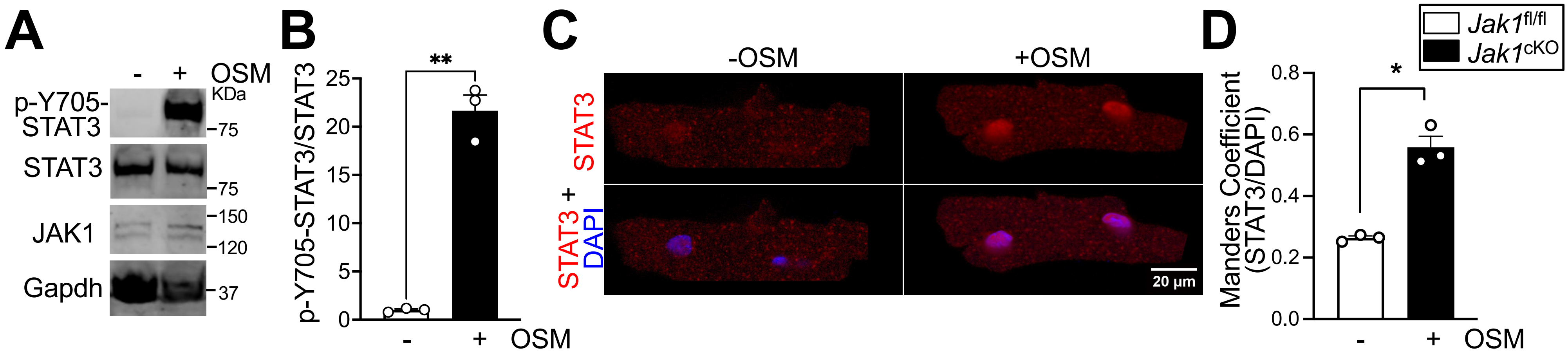

Supplemental Figure S1. Cytokine-dependent STAT3 signaling is preserved in *Myh6*-Cre transgenic cardiomyocytes. (A) Adult ventricular cardiomyocytes (ACMs) were isolated from male and female *Myh6*-Cre control hearts at 2 months of age and treated with vehicle or oncostatin-M (OSM, 10 ng/mL for 30 minutes) before harvesting for Western blotting or fixing for immunocytochemistry to assess STAT3 phosphorylation and nuclear translocation, respectively. (A) Western blotting and (B) quantification of STAT3 phosphorylation at Tyr-705 normalized to total STAT3. n=3. (C) Representative images of immunostaining for endogenous STAT3 (red) in *Myh6*-Cre ACMs with or without OSM treatment. Nuclei were stained blue with DAPI. Scale bar = 20 µm. (D) Manders coefficient for colocalization of STAT3 with nuclear DAPI signal in ACMs of the indicated treatment. n=3 with 35-60 ACMs analyzed per biological replicate. Data are presented as the mean value ± the standard error of the mean. *P<0.05, **P<0.01, paired t-test. Related to Figure 2.

Fig S2

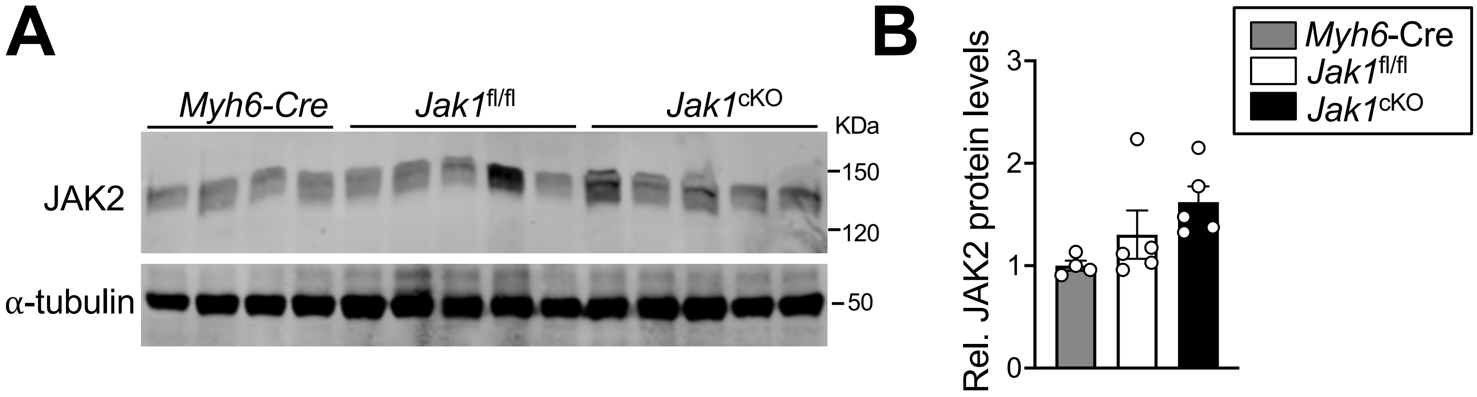

Supplemental Figure S2. JAK2 protein levels are not altered in hearts of mice with cardiomyocyte-specific loss of JAK1. (A) Western blotting and (B) quantification of JAK2 protein levels relative to α-tubulin in hearts of the indicated genotypes of male and female mice at 6 months of age. n=4 *Myh6*-Cre, 5 *Jak1*$^{fl/fl}$, and 5 *Jak1*^{cKO}. Data are presented as the mean value ± the standard error of the mean.

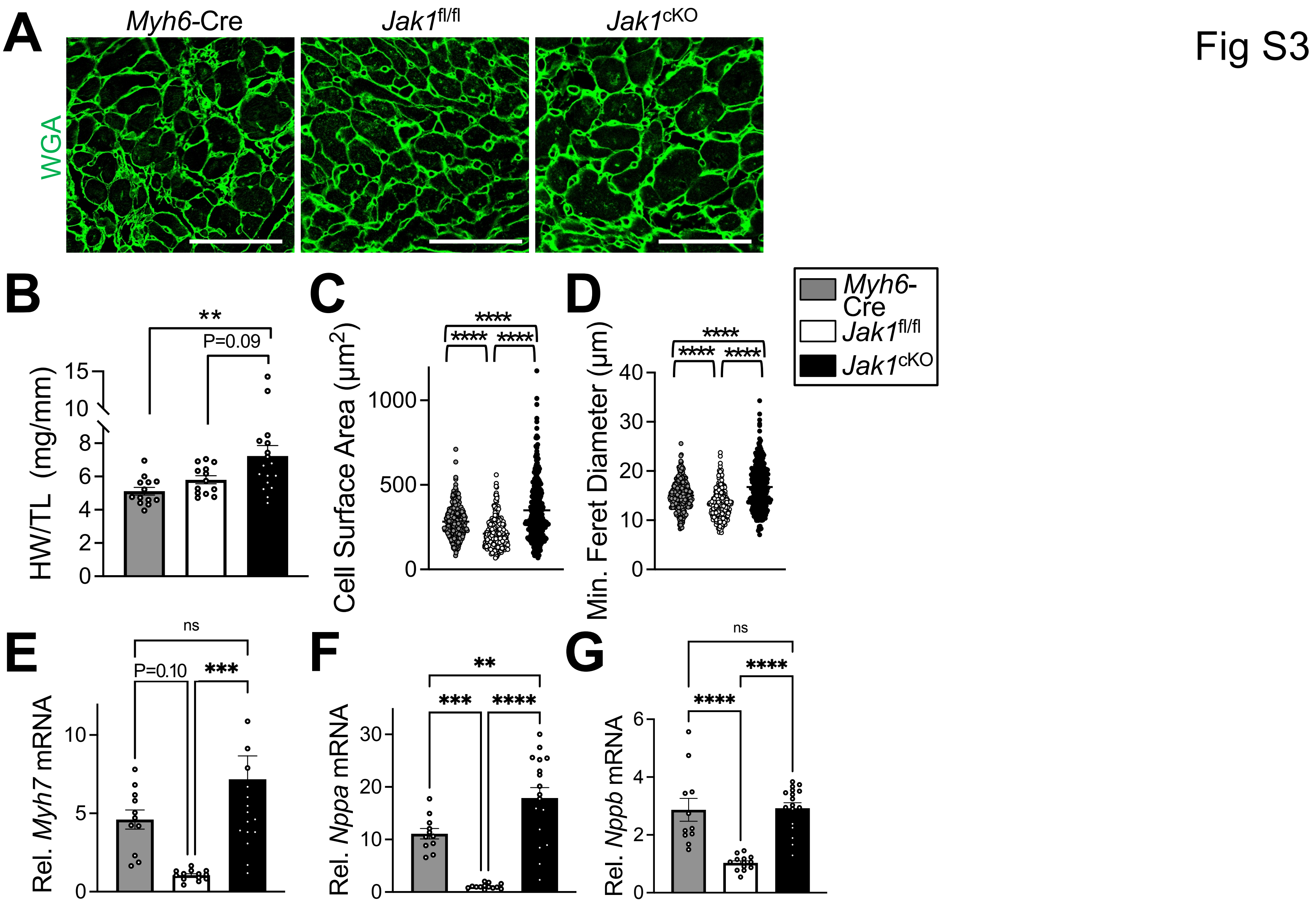

Supplemental Figure S3. Hypertrophic growth in *Myh6*-Cre transgenic control and *Jak1*^{cKO} hearts. (A) Wheat germ agglutinin (WGA)-staining of cardiac sections (scale bar = 100 µm) of female mice and (B) heart weight-to-tibia length (HW/TL) ratios (n=13 *Myh6*-Cre, 13 *Jak1*$^{fl/fl}$, and 17 *Jak1*^{cKO}) and quantification of (C) cardiomyocyte cell surface area and (D) minimum Feret's diameter from cardiac sections of the indicated genotypes of male and female mice at 8 months of age. n=300 cardiomyocytes evaluated per genotype (60 cells per heart from five animals per genotype) in C and D. (E-G) Hypertrophic marker gene expression. mRNA levels of the pathological hypertrophic marker genes (E) *Myh7*, (F) *Nppa* (ANP), and (G) *Nppb* (BNP) were quantified by qPCR in 8 months old male and female control and *Jak1* cardiomyocyte-deleted hearts. n=11 *Myh6*-Cre, 13 *Jak1*$^{fl/fl}$, and 17 *Jak1*^{cKO}. Data in B and E-G are presented as the mean value ± the standard error of the mean. **P<0.01, ***P<0.001, ****P<0.0001, *ns,* not significant, one-way ANOVA with Tukey's multiple comparisons test. Related to Figure 5A and B.

Fig S4

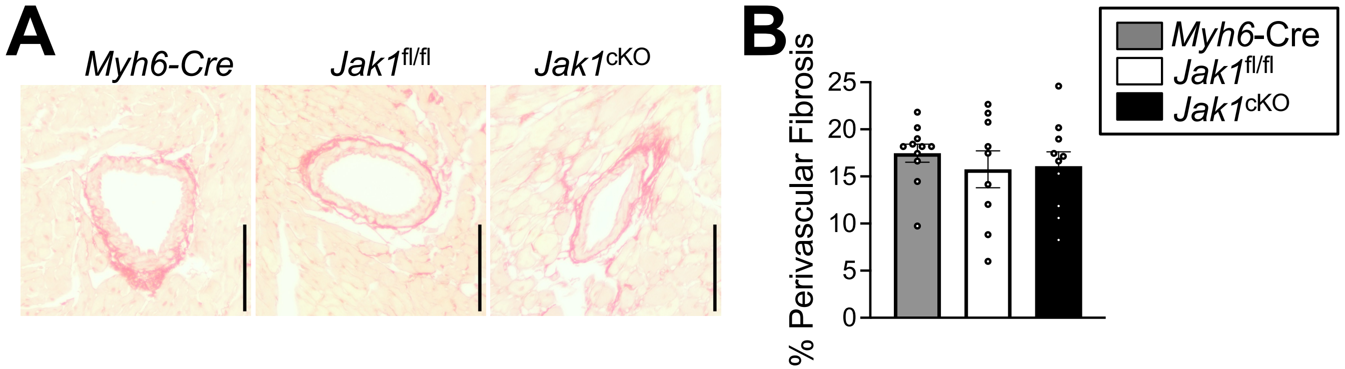

Supplemental Figure S4. Perivascular fibrosis is not significantly changed in the aged heart by cardiomyocyte loss of JAK1. (A) Representative images of Picro Sirius Red-stained cardiac sections (scale bar = 100 µm) and (B) quantification of perivascular fibrosis in both male and female mice lacking cardiomyocyte Jak1 (*Jak1*^{cKO}) compared to controls (*Myh6*-Cre and *Jak1*$^{fl/fl}$) at 8 months of age. n=11 *Myh6*-Cre, 9 *Jak1*$^{fl/fl}$, and 10 *Jak1*^{cKO}. Representative images in A are mixed sexes (*Myh6*-Cre and *Jak1*$^{fl/f}$ female, *Jak1*^{cKO} male). Data are presented as the mean value ± the standard error of the mean. Related to Figure 5C and D.

Fig S5

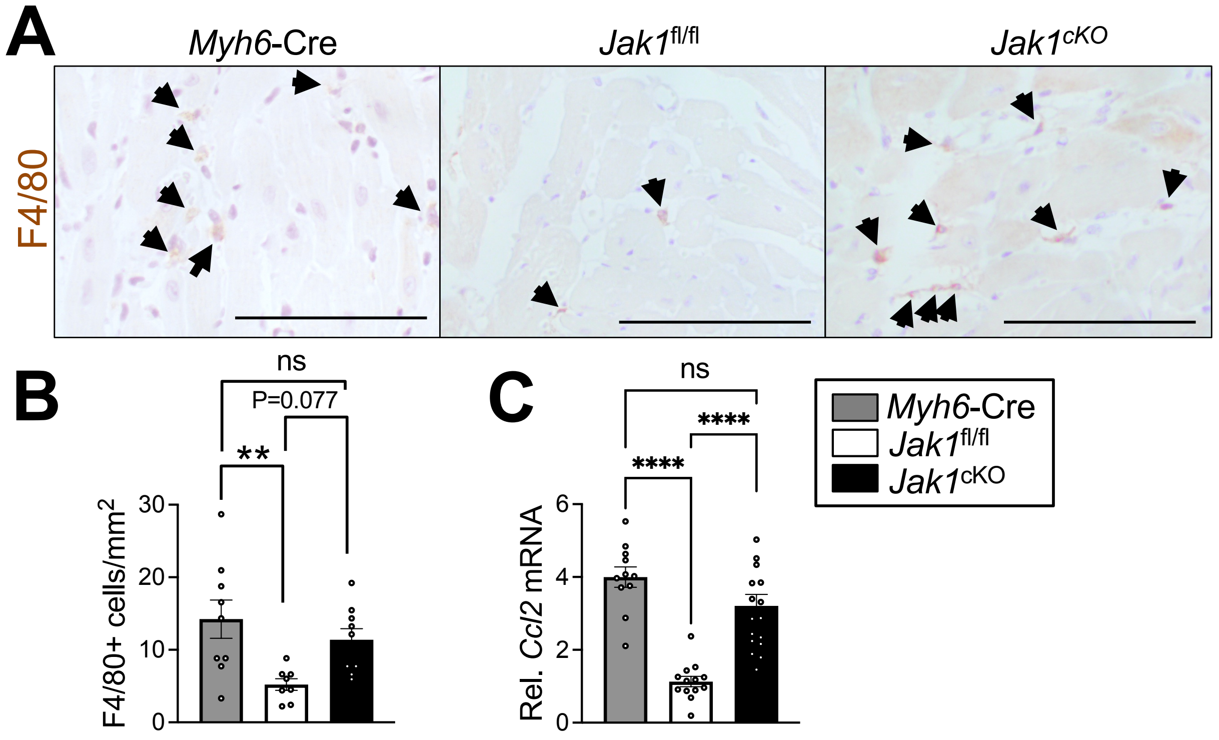

Supplemental Figure S5. Myocardial inflammation in *Myh6*-Cre transgenic control and *Jak1*^{cKO} mice. (A) Representative images of F4/80 immunohistochemistry in male mice and (B) quantification of F4/80 positive cells from cardiac sections of male and female mice of the indicated genotypes at 8 months of age. Brown HRP signal (arrowheads) indicate F4/80+ cells in A. Scale bar = 100 µm. n=9 *Myh6*-Cre, 8 *Jak1*$^{fl/fl}$, and 9 *Jak1*^{cKO}. (C) mRNA levels of the chemokine *Ccl2* (MCP-1) were quantified by qPCR in 8 months old male and female mice of the indicated genotypes. n=11 *Myh6*-Cre, 13 *Jak1*$^{fl/fl}$, and 17 *Jak1*^{cKO}. Data are presented as the mean value ± the standard error of the mean. **P<0.01, ****P<0.0001, *ns,* not significant, one-way ANOVA with Tukey's multiple comparisons test.
